# Quantitative identification of technological paradigm changes using knowledge persistence

**DOI:** 10.1371/journal.pone.0220819

**Published:** 2019-08-15

**Authors:** Changbae Mun, Sejun Yoon, Yongmin Kim, Nagarajan Raghavan, Hyunseok Park

**Affiliations:** 1 Department of Information Systems, Hanyang University, Seoul, Republic of Korea; 2 Engineering Product Development (EPD) Pillar, Singapore University of Technology and Design (SUTD), Singapore; IMT Institute for Advanced Studies Lucca, ITALY

## Abstract

This paper proposes a method to quantitatively identify the changes of technological paradigm over time. Specifically, the method identifies previous paradigms and predicts future paradigms by analyzing a patent citation-based knowledge network. The technological paradigm can be considered as dominantly important knowledge in a specific period. Therefore, we adopted the knowledge persistence which can quantify technological impact of an invention to recent technologies in a knowledge network. High knowledge persistence patents are dominant or paradigmatic inventions in a specific period and so changes of top knowledge persistence patents over time can show paradigm shifts. Moreover, since knowledge persistence of paradigmatic inventions are increasing dramatically faster than other ordinary inventions, recent patents having similar increasing trends in knowledge persistence with previous paradigms are identified as future paradigm inventions. We conducted an empirical case study using patents related to the genome sequencing technology. The results show that the identified previous paradigms are widely recognized as critical inventions in the domain by other studies and the identified future paradigms are also qualitatively significant inventions as promising technologies.

## Introduction

Technological paradigm and trajectories have been recognized as an important framework to understand evolutionary processes and underlying knowledge structures of a technological domain (TD) [1–6]. Technological paradigm is defined to be a set of principles, search heuristics and specific knowledge base which are dominant and pervasive in the TD [5–10]. Once a paradigm is established, the direction of future developments, technological trajectory, is programmed [5–10]. The emergence of a new technological paradigm providing much better performance than the previous paradigm alters the whole structure and mechanism in the TD and provides new knowledge framework for problem-solving in technological trajectories, which is called a paradigm shift [11–13]. There has been much effort to objectively identify main developmental paths for tracing technological trajectories using a patent citation network [14–17]. However, few studies to objectively identify technological paradigms have been conducted, because it is fundamentally difficult to quantitatively define and identify the dominant technological knowledge base and its changes in terms of qualitative characteristics of technological paradigms.

This paper proposes a quantitative approach to identify technological paradigms and dynamic changes of them. Instead of directly identifying dominant technological knowledge for a specific period, this study identifies one or some paradigmatic inventions dominantly influencing on the recent technological developments as the underlying knowledge for a specific time period, and the dynamic changes of paradigmatic inventions over time are considered as technological paradigm shifts. Specifically, we adopted a knowledge persistence measurement, suggested by [18], that quantifies how much knowledge of an invention contributes to recent inventions in a patent citation-based knowledge network. Therefore, high persistence patents (HPPs) in a specific period can be considered as technological paradigms.

Given that the analytic purpose of paradigm identifications is basically toward to the future-oriented strategies, objective identification of future paradigms seems to be a significant intelligence for the purpose. Knowledge persistence is basically a patent citation-based metric and so, similar to other patent citation-based approaches, earlier patents usually have higher knowledge persistence than recent patents. Direct use of knowledge persistence seems to be inappropriate to identify future paradigms. To overcome this, we developed a new metric, named the potential paradigm (PP), based on the increasing trend in knowledge persistence of previous technological paradigms, which identifies the patents recently invented and so having relatively low knowledge persistence, but high possibility to become the HPPs.

To test the method, this paper conducted an empirical case study using the genome sequencing technology; we selected the genome sequencing because it has highly complex knowledge structure and quantitative technological analysis is useful to investigate the TD. The results show that the proposed method can identify patents widely recognized as paradigmatic inventions in different time periods. In addition, some recent patents, not HPPs but paid great attention in the TD, are objectively identified by PP. In particular, all previous paradigmatic inventions were also identified by PP as future paradigms in previous timeframe, the PP seems to be a reliable metric to predict future paradigms. Therefore, recent patents listed in Top PP patents can be considered as potential future paradigms and our qualitative analysis supports the results as well.

The rest of this paper is structured as follows: Section 2 reviews the concept of knowledge persistence, Section 3 describes the proposed method, Section 4 presents the empirical analysis and discussion of the results, and finally conclusion is drawn in Section 5.

## Knowledge persistence

The knowledge persistence measurement, proposed by [18], is a metric to find technologically important knowledge in a specific TD by quantifying the persistent knowledge of a patent. The underlying idea of the method is a knowledge recombination process for creating new knowledge. Since every invention is basically developed based on prior knowledge, some proportion of prior knowledge are adopted to new inventions and the inherited knowledge is continually conveyed to later inventions by the same process.

**Fig 1:**
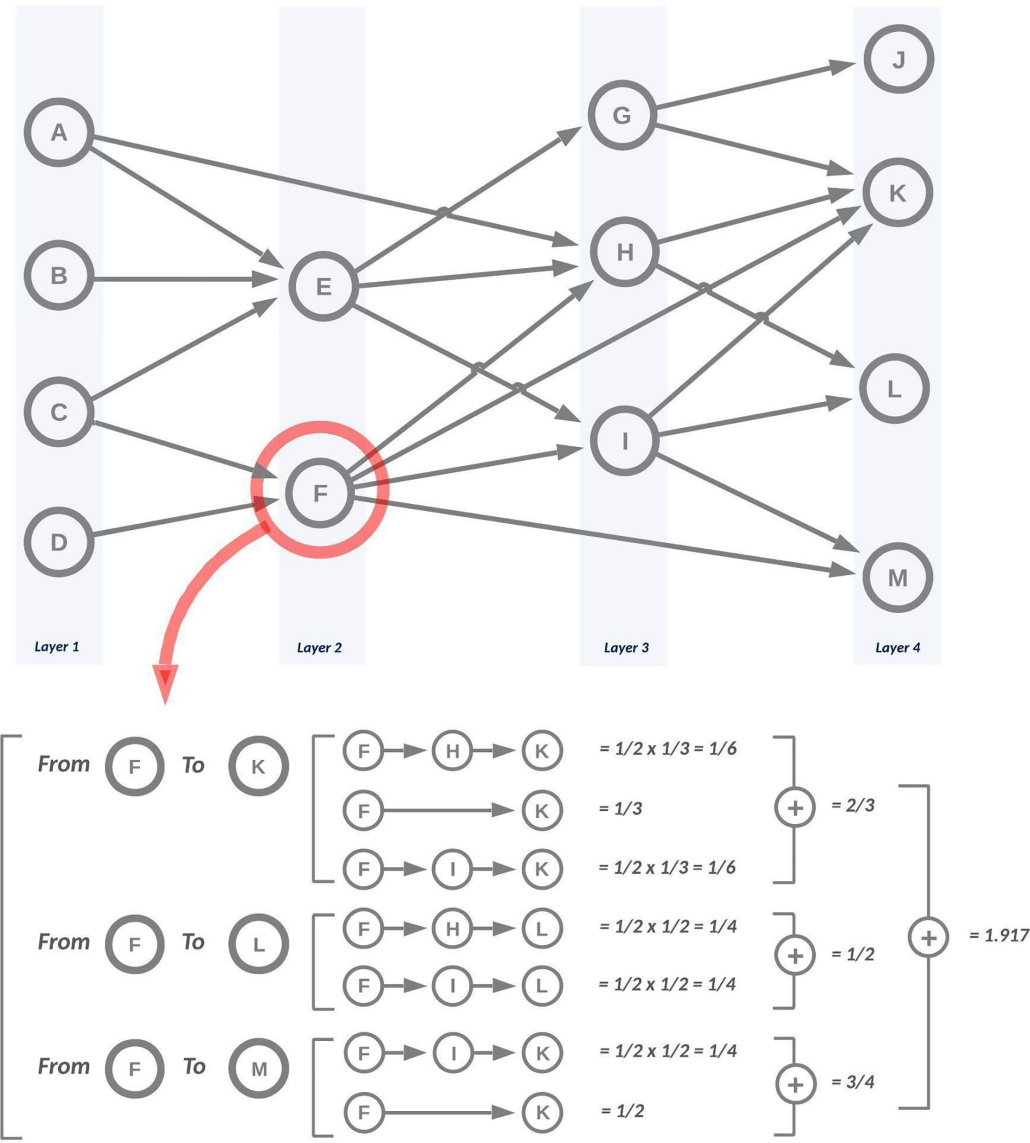
Knowledge persistence measurement, redrawn from [15]. Note: layers represent the overall structure of knowledge flows. The number of layers is the longest sequences of citation link from end-points to start-points. The inherited knowledge is calculated based on the number of knowledge sources. For example, patent F cites patent C and D, so patent F inherits 1/2 of its knowledge respectively from patent C and D.

In patent system, the relationship between citing and cited patents is widely used as a proxy for knowledge flows; even though some studies criticize the use of citations as knowledge flows [19], there is little objective evidence to support these criticisms [20] and most of studies using patent citations deem ‘citations as knowledge flows’. The citing patent (as a new invention) is invented based on the proportion of knowledge of the cited patents [21–26], So the number and structure of backward citations of a patent can be used to calculate how much knowledge is inherited from the cited patents by the focal patent. Fig 1 shows how knowledge persistence of a patent is quantitatively calculated in a patent citation network.

Major difference of genetic knowledge persistence from other citation-based approaches is that it can consider not only the local characteristic of a patent derived by direct citations, but also the global characteristics derived by indirect citations. Park and Magee [27] tested the performance of some citation-based global and local metrics by a simulation and empirical analysis, and the results show that global approaches provide better performance in identification of technological discontinuities than local approaches. Therefore, the knowledge persistence can be used to identify technologically important patents that dominantly impact on the recent technological developments from the global perspective [18] and so use of knowledge persistence to identify technological paradigm in a specific period seems to be suitable.

There are two important issues in use of the genetic knowledge persistence. First, a different set of patents might produce very different knowledge persistence for the same patent. For instance, if a patent set does not include some important descendant patents of a patent, which are cited by many later patents, knowledge persistence value of the focal patent in the patent set can be far lower than value in different sets. Therefore, a set of patents for a specific TD should be carefully collected and this is why we adopt Classification Overlap Method (COM), suggested by [28, 29]. Second, since knowledge persistence is basically a citation-based approach, earlier patents usually have higher knowledge persistence than recent patents, i.e. time effect. Knowledge persistence might not be appropriate to find future paradigms. To overcome this, this paper developed a new metric (PP) that can identify potential candidates of future paradigms. It will be drawn in Section 3.

## Method

### Identification of patents related to specific technological domain

In order to analyze patents for technological research, it is basically important to isolate the patents highly relevant to the TD. Depending on the accuracy of a set of patents, even the same methodology can produce very different results. This paper adopted the COM to collect a set of patents for a specific TD [28, 29]. We deem the meaning of TD is a set of technologies that fulfill specific generic function using a specific scientific effect [30] and COM provides a reliable performance on the patent collection, an average patent relevancy for 44 TDs is 85.54% [31, 32]. COM identifies the TD-relevant patents based on the characteristics of co-classification to multiple patent classification systems. Specifically, patents classified into specific IPCs (International Patent Classification) and UPCs (United States Patent Classification) at the same time can represent a specific TD [28]. For example, the combination of IPC F03D (Wind motors) and UPC 290 (Prime-mover dynamo plants) and 416 (Fluid reaction surfaces) isolates the patents related to the Wind turbine technology with 94% accuracy [33]. The way to find a specific combination of IPCs and UPCs for a specific TD is as follows. First, collection of patents of interests by pre-search using the representative keywords of a TD, Second, identifying most representative IPCs (4-digit sub-class level) and UPCs (3-digit class level), which show high recall *(# patents in the pre-search within the patent class/# collected patents in the pre-search*) and precision *(# patents in the pre-search within the patent class/# patents in patent class*) and so high mean-precision-recall (*(precision + recall)/2*). Third, the combinations of IPCs and UPCs having high mean-precision-recall are identified and the relevancy of the included patents in the overlapped classes is checked to determine the combination.

### Measurement of genetic knowledge persistence over time

The knowledge persistence of a patent is determined based on the topological position in a patent citation network. Specifically, the number of descendants and generation length of a specific patent are directly related to the degree of knowledge persistence. The first step, therefore, should be the identification of the lineage structure of a TD by identifying the longest sequence of patent citations in a patent citation network. We call the knowledge generation of a TD as the ‘layer’ and all patents are mapped on a specific layer by a backward tracing from patents on the last layer, which do not have any forward citations. The proportion of inherited knowledge from an ancestor patent to a descendant patent is measured by 1/the number of backward citations of the descendant patent. For example, if patent A is cited by two patents (B and C), and the number of backward citations of B are two and of C are four, the inherited knowledge of A in B is 0.5 and C is 0.25. Therefore, knowledge persistence of a patent in a patent citation network can be expressed as the following equation [15, 27]:

$$Persistence\;of\;patent\;A=\sum_{i=1}^{P}\sum_{j=1}^{Q_{i}}\prod_{k=1}^{R_{j}-1}\frac{1}{Bwd({Patent}_{ijk})},$$

where *P* means the number of patents that cites patent *A* directly or indirectly on the last layer. *Q*_*i*_ means all paths available from the patent *i* in the last layer to the patent *A*. *R*_*j*_ means the number of patents which exist in the *j-th* path from patent *i* to the patent *A*. *Patent*_*ijk*_ means *k-th* patent in the *j-th* path from patent *i* to patent *A*. *Bwd(Patent*_*ijk*_*)* means the backward citation index of *Patent*_*ijk*_ after the link between patents from the first layers to the right previous layer that includes *Patent*_*ijk*_ is deleted.

**Fig 2:**
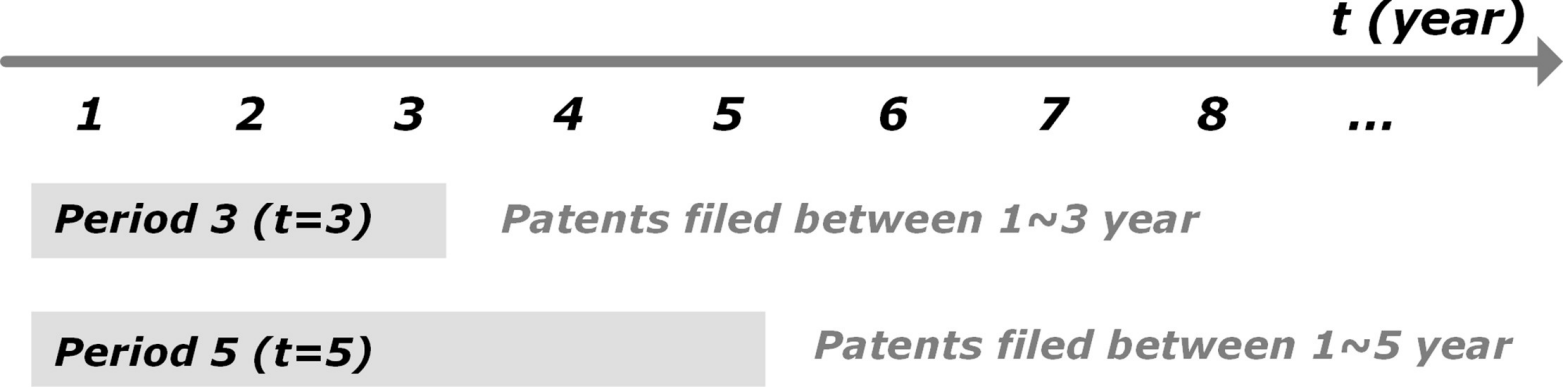
Patents in each cumulative time interval.

In order to analyze dynamic changes of knowledge persistence over time, knowledge persistence of each patent is calculated in every time period (from the first to *t-th* year) by using patents included in the period (Fig 2). For example, if whole timeframe of a patent set is five years, knowledge persistence of a patent filed in the first year is calculated five times and a patent filed in the third year is calculated three times.

### Identification of previous paradigms

**Fig 3:**
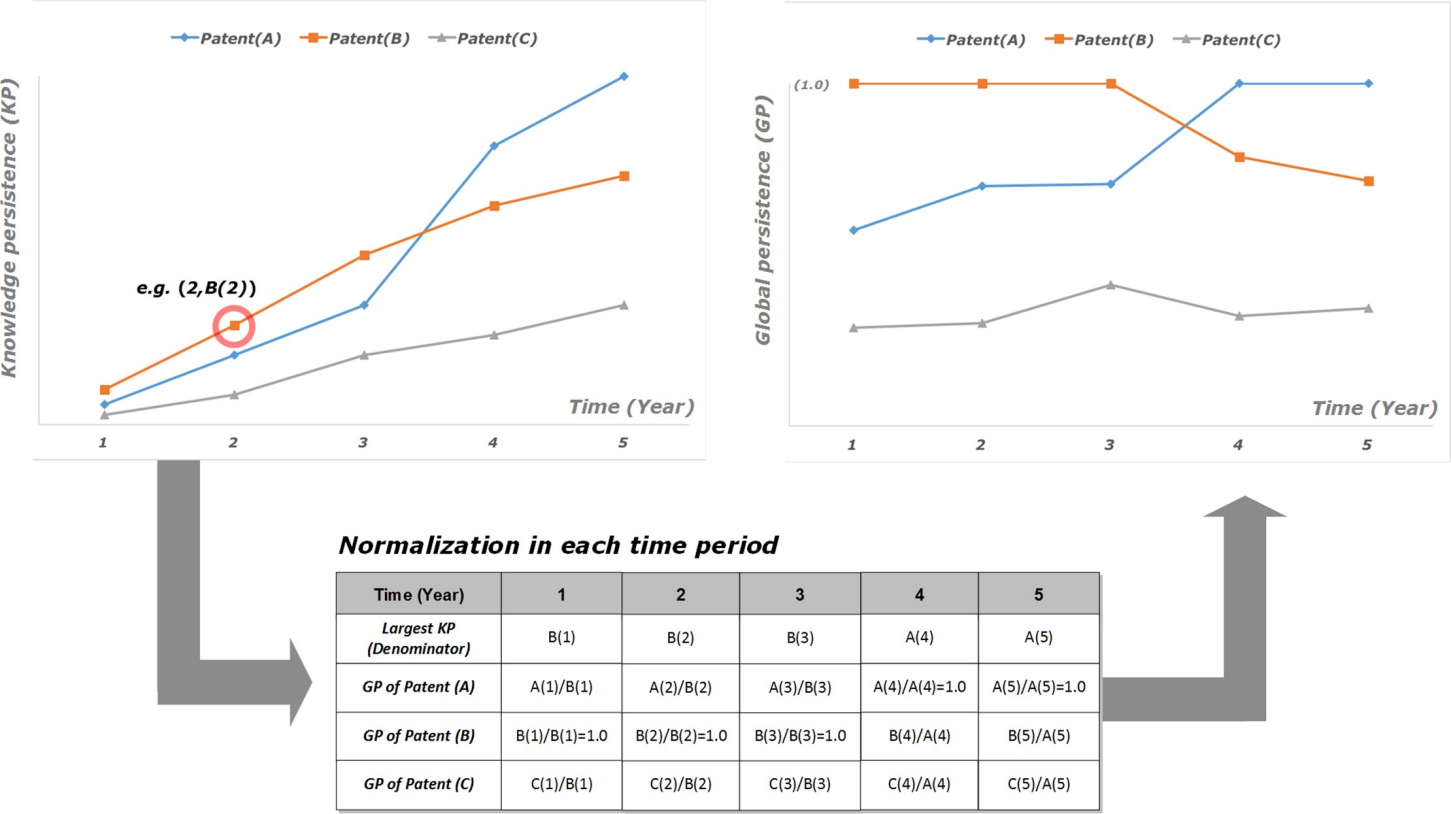
Normalization of knowledge persistence in each time period.

Based on the calculated knowledge persistence in the sequence of time periods, previous technological paradigms are identified. The patents having distinctively high knowledge persistence in a specific period can be represented as the technological paradigms in the period and the changes of top knowledge persistence patents over time show paradigm shifts. The patents included in a specific period are normalized by dividing the largest knowledge persistence in the period to transform the range to [0, 1], we call the normalized value as the global persistence (GP) (Fig 3).

In order to identify previous technological paradigms, the following two issues have to be considered. First, even though patents whose GP = 1.0 are a clear signal for paradigms, other high GP patents whose GP is less than 1.0 but very higher than other ordinary patents might have important knowledge to represent or support technological paradigm in a specific period. Therefore, it would be better to set the cut-off value not to miss other non-ignorable inventions. Since knowledge persistence in a specific TD shows power-law distribution [27], only small number of significant patents can have high GP; our tests show that even low cut-off (GP>0.3) identifies less than 30 patents out of 5,000~30,000 patents. This paper sets the cut-off value to be 0.7 and so the patents whose GP> = 0.7 are considered as the previous technological paradigms. Second, some early patents whose GP are very high in their early years but extremely falls over time should not be considered as the previous paradigms. This case is usually caused by the small sized patent set in early time period. If the number of patents in a patent set is not enough, there are almost no difference among top patents in knowledge persistence. Therefore, top GP patents in early years but rapidly falling down after the peaks are not technological paradigms, and so we set one more cut-off (GP<0.2) to ignore the mentioned patents. Therefore, paradigmatic patents are GP> = 0.7 in any time period and, at the same time, GP> = 0.2 after their peaks.

**Fig 4:**
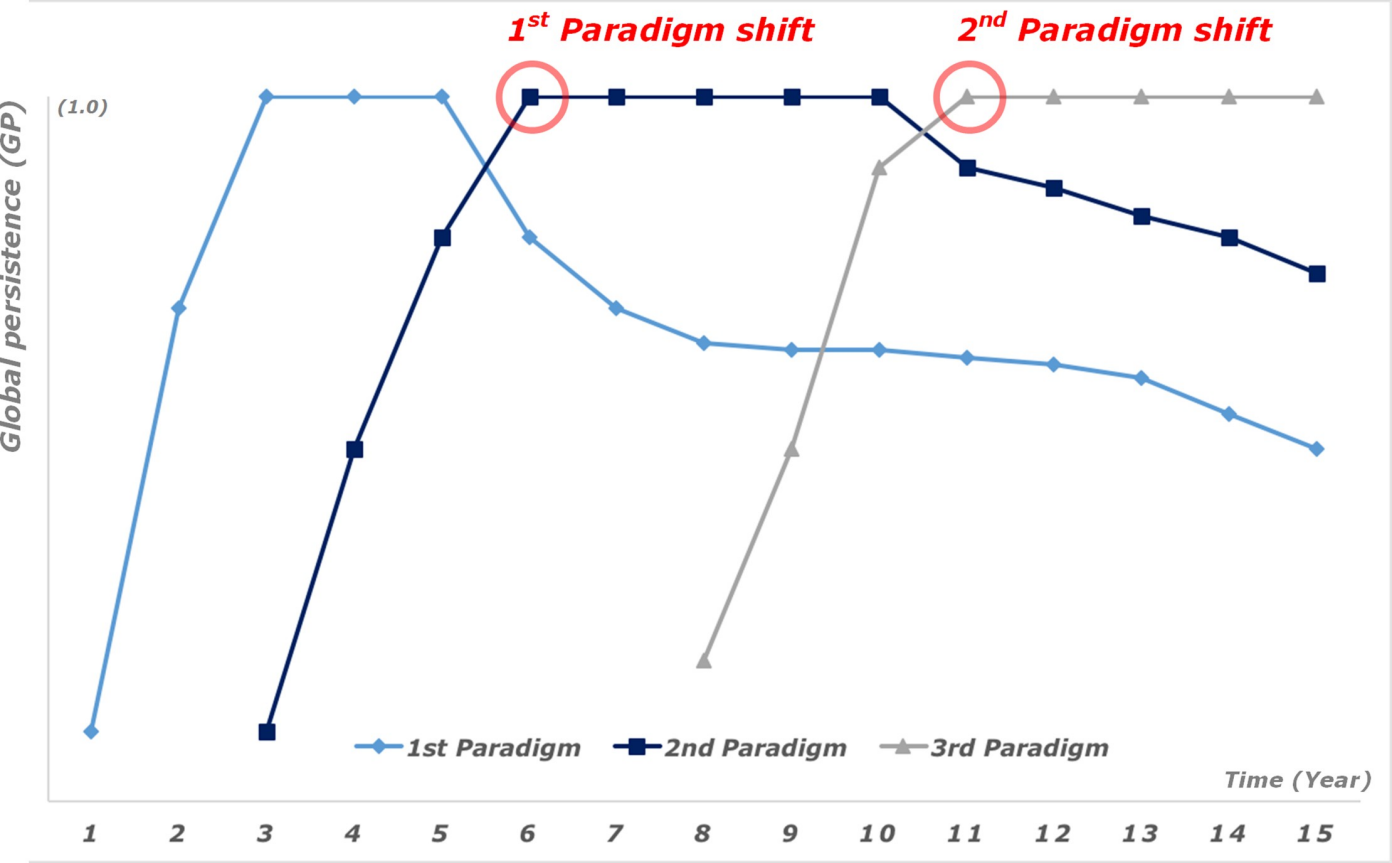
Paradigm shifts on GP vs. time graph.

Since technological paradigms change over time, even though some TD might show very slow evolution trends, GP of patents are also changes over time. Some high GP patents fall down and some emerging patents increase and replace the position of high GP. This trend represents the paradigm shifts and Fig 4 shows the paradigm shifts in the GP vs. year graph.

### Identification of future paradigms

**Fig 5:**
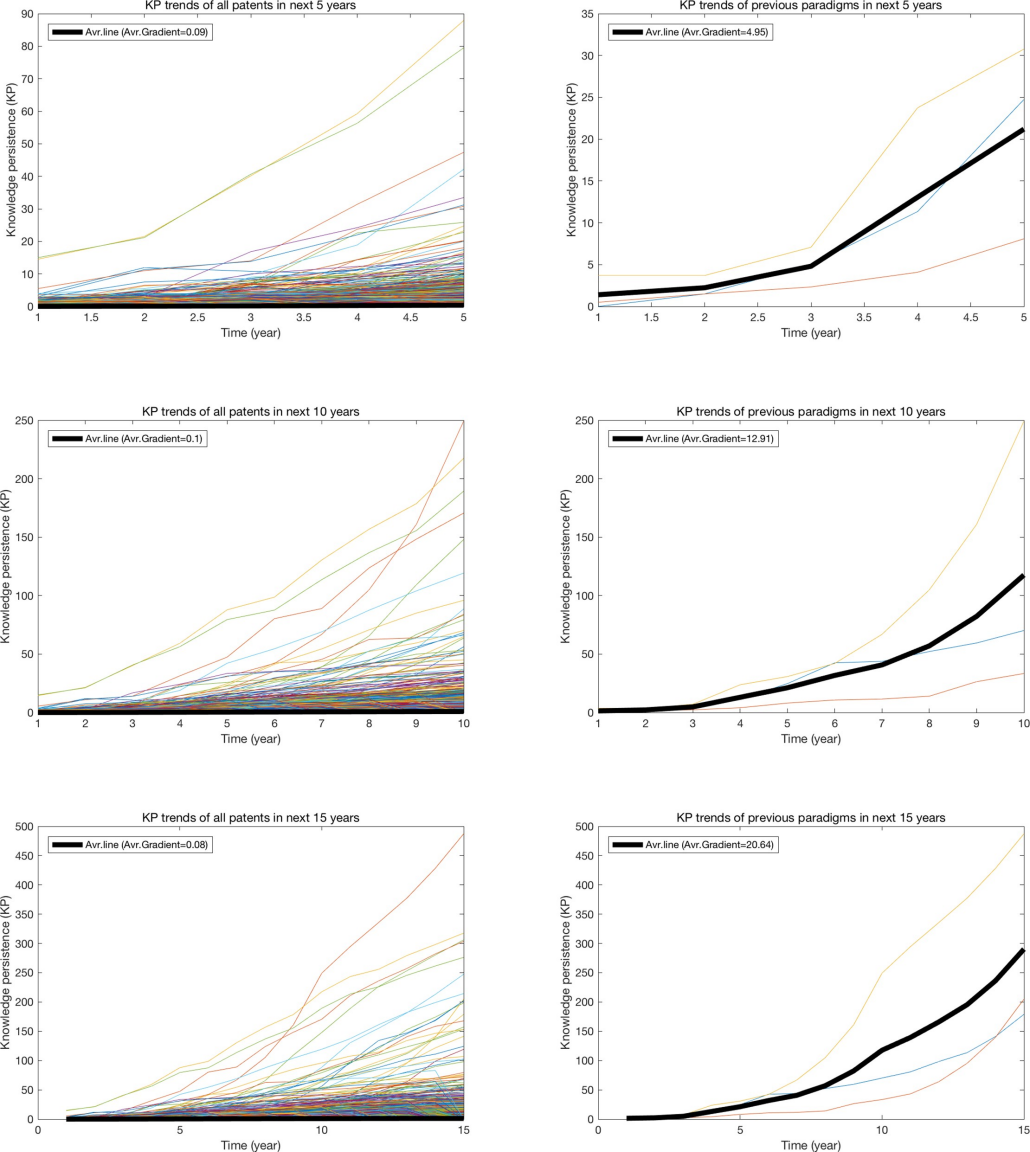
Increasing trends of knowledge persistence over time in genome sequencing technology in different time period (next 5, 10, and 15 years). *Notes*: Left graphs are about KP trends of all patents and right graphs are about KP trends of previous paradigm patents. All patents are rearranged in the 0 year and average KP over time is also visualized by black thick-line.

As we mentioned in the section 2.2, recent patents are basically difficult to obtain high knowledge persistence than older patents due to the time-effect. This means that knowledge persistence might be difficult to identify the recent inventions having high potential to become future paradigms. In order to identify the candidates of future paradigms, we developed a new metric by analyzing the increasing trend in knowledge persistence of previous paradigms. We found that the knowledge persistence of previous paradigms has increased significantly faster than other patents in whole time periods (Fig 5). In particular, increasing rates in knowledge persistence of the previous paradigms by calculating patents within first a few years are far larger than other ordinary patents, and most patents rapidly increasing in first few years (e.g. 5 years) still increase faster than other patents in longer periods (e.g. 10 or 15 years). Therefore, a gradient in knowledge persistence vs. time graph can be a key indicator to identify the candidates of future paradigms and even short period of time can provide a good performance in identification.

**Fig 6:**
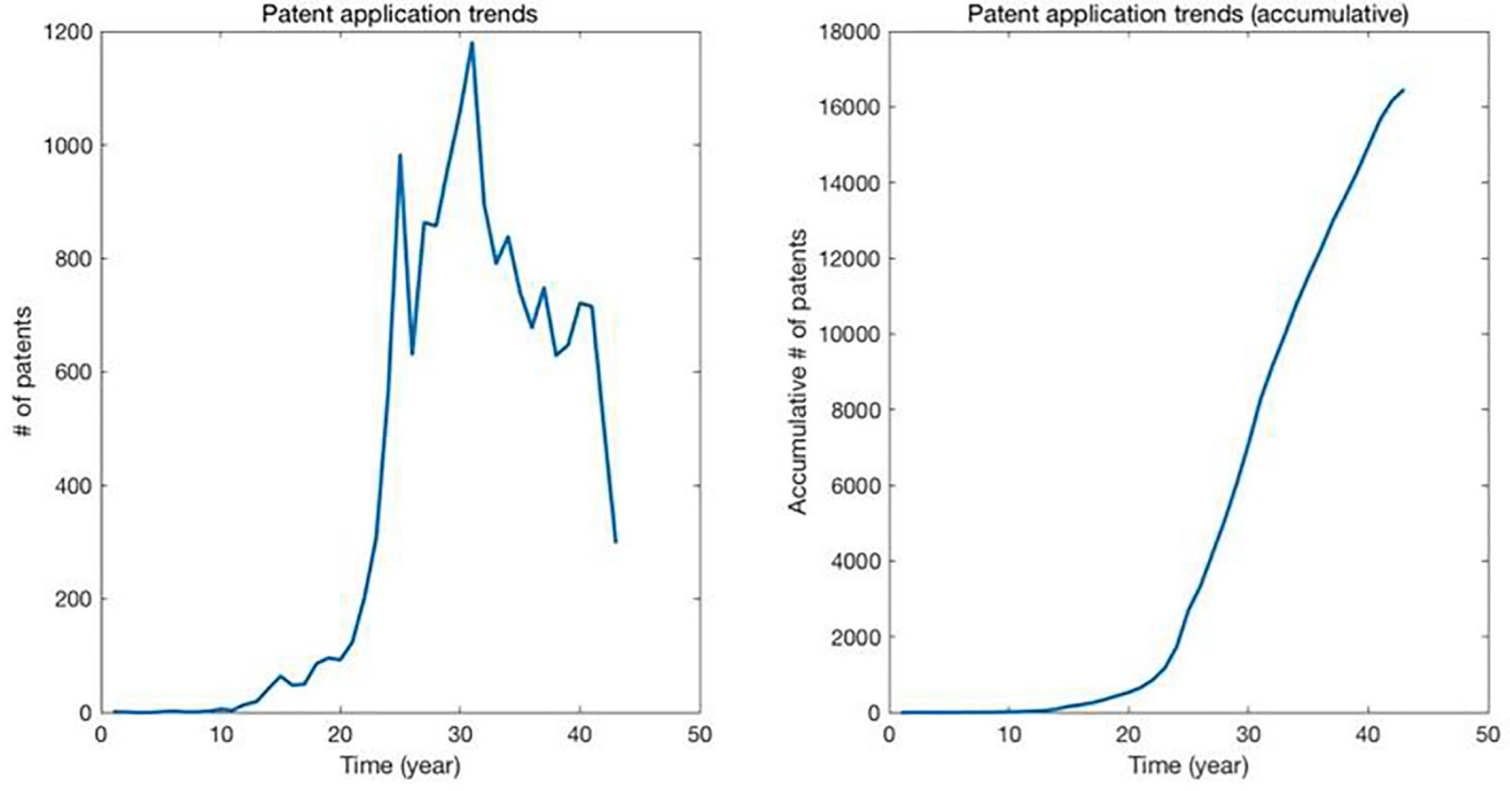
Patent application trends in Genome sequencing domain.

However, given that the patent application trend in a specific TD is inconstant over time (Fig 6), the metric for future paradigm identification should include a size normalization of patent set to reduce the possibility of the size effect. For example, if the gradient of two patents (A and B) filed in different years is same, but the number of patents included in next *N* years of A is far larger than B, the patent B should be evaluated as the more promising candidates, because the patent B inherits its knowledge to higher proportion of descendants in the given period. Therefore, we defined the metric, Potential Paradigm (PP), to identify future paradigm as follows:

$$Potential\;Paradigm(PP)\;of\;Pat(i)=\frac{Average\;gradient\;in\;next\;N\;years}{Size\;of\;patents\;in\;next\;N\;years}=\frac{\left(\frac{{{KP}_{t+N}-KP}_{t+1}}{{Year}_{t+N}-{Year}_{t+1}}\right)}{ln(NumPat)},$$

where *Pat(i)* is the *i-th* patent in the TD, *t* is application year of *Pat(i)* and *Avrerage gradient in next N year* is the average gradient of knowledge persistence within the next *N* years, specifically it is calculated by the knowledge persistence (KP) in the *t+N* year (*KP*_*t+N*_)–knowledge persistence in the *t+1* year (*KP*_*t+1*_) over the the last year (*Year*_*t+N*_)–first year (*Year*_*t+1*_), and *NumPat* is the number of patents in the next N years and we applied a logarithmic transformation to reduce the skewness by very large number of patents in specific periods.

The empirical case in Section 4 will show that all of previous paradigms are identified by the metric (PP) and so the relatively new patents listed in the top-ranking can be considered as the candidates of future paradigms.

## Empirical analysis

### Introduction to Genome sequencing technology

Genome sequencing is a process to determine DNA sequence of an organism’s genome. The first genome sequencing technology is widely recognized as the Sanger sequencing (or dideoxy chain-termination method), developed by Frederick Sanger and his colleagues in 1977 [34]. The mechanism of this approach is fundamentally based on a primer-extension strategy and so it amplifies DNA sample using PCR (polymerase chain reaction) and identifies genome sequences by using acid and base reaction. Even though the Sanger sequencing requires large amount of time and cost for analysis and so it is inappropriate for a large-scale DNA sequencing, it is relatively easy to use and provides high accuracy. Since its advantages, the method was most widely adopted method for 40 years and is still the basis of many of recent DNA sequencing systems [35]. The next breakthrough in DNA sequencing was mostly related to a high-throughput automated DNA sequencing, actively developed in the mid to late 1990s. The second-generation technologies are fundamentally based on the concept of the Sanger’s approach, but provide a significantly faster sequencing by analyzing the massive amount of genome sequences in parallel or introducing fluorescent dyes and capillary electrophoresis into the process [35]. Recently, there are many new DNA sequencing approaches, such as single molecule real time sequencing, sequencing-by-synthesis and ion torrent sequencing. They are basically based on the different concepts from previous approaches and mainly focusing on high-throughput sequencing with low cost and high accuracy. However, most of recent approaches are still in the laboratory research phase and so the second-generation DNA sequencing related patents are dominant in the TD.

### Data

Total 16,468 US granted patents related to genome sequencing technology were collected from the PatSnap(www.patsnap.com). The search query is formulated based on the COM and scope of the data is from 1971.01.01 to 2013.12.31 (Table 1). To simplify this time scale, we transformed the whole years into from 1 to 43, i.e. minus 1970 from original year.

**Table 1:** Summary of data.

Search query	Number of patents	Range/scope
C12Q and (435/6.11 or 435/6.12 or 536/24.3)	16,468	US granted patents from 1971.01.01 to 2013.12.31 (application date)

### Result

#### Previous paradigms

**Fig 7:**
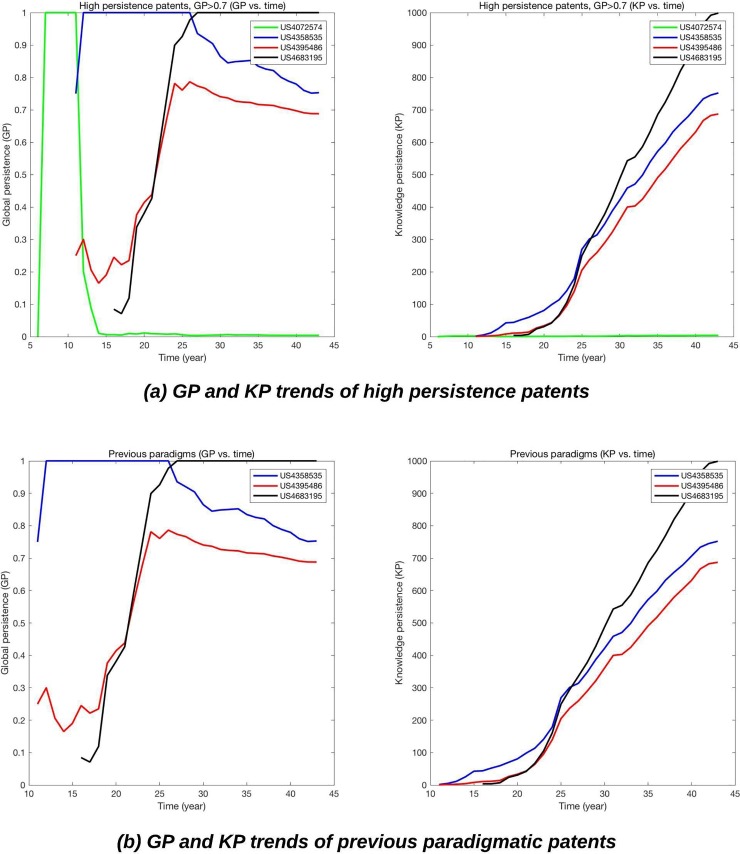
Dynamic changes in KP and GP of previous paradigms.

We identified three patents as the previous paradigms—US 4358535, US 4395486, and US 4683195; as shown in Fig 7(A), if the second rule (GP> = 0.2 after the peak) is not applied, patents like US 4072574 might be inappropriately identified as paradigms. In GP vs. time graph (left image in Fig 7(B)), US 4358535 and US 4683195 are two the most important paradigmatic inventions (GP = 1.0) during whole period. As we expected, all identified paradigms are related to the second-generation DNA sequencing, because most of first-generation technologies, i.e. Sanger sequencing, were not patented [35].

US 4358535, the first paradigm in the TD, is an invention that aims at detection of pathogen in medical sample. This invention puts the nucleotide probe that has DNA sequence complementary to the sequence of pathogen on a sample and identifies the presence of pathogen through heterogeneous hybridization assay that visually shows combination of pathogen and complementary DNA. The nucleotide probe can be radioisotopes, ligands, phosphors, chemical derivatives, enzymes, or antibodies. The patent is significant because it broadens the coverage of use of DNA probes for diagnostic purposes [36].

US 4683195 is a patent directed to a process for amplifying and detecting nucleic acid sequence of target-samples. As shown in the left graph of Fig 7(B), this patent achieved the highest GP from 1997 (27^th^ year), and became the 2^nd^ paradigmatic invention in the TD. This invention provides much more efficient and faster sequencing by utilizing a repetitive reaction to accomplish the amplification of the desired nucleic acid sequence. There are some studies that support the importance of the patent. Aida, Konishi [37] selected US 4683195 as one of pioneer patents in the field of biotech and Arts and Veugelers [38] identified the patent as the one of breakthrough invention in the PCR technology.

US 4395486, even though this patent cannot reach at GP = 1.0, also seems to be one of dominant invention in the TD. The invention is a technology that diagnoses two pairs of nucleic acid in DNA-extracted solution through restriction enzymes and identify basic nucleotide sequence ‘CTNAG’ of DNA fragment to detect DNA that causes ‘Sickle cell anemia’. As shown in the right image of Fig 7(B), the patent was initially not paid attention in its early phase, but became one of the most important knowledge in the TD after quite a long time.

Interestingly, Bruck, Réthy [39] quantitatively identified the most significant inventions from all granted patents between 1976 and 2012 (almost 3.93 million patents) and their result shows that our identified previous paradigmatic patents are ranked in the Top 10 inventions (US 4358535 is ranking #9, US 4395486 is ranking #4, and US 4683195 is ranking #1) out of 3.93 million inventions. This result also can strongly support the reliability of our method and results.

#### Future paradigms

We first calculated PP of each patent by using all other patents involved in the next 5, 10, and 15 years to show that how long time the metric needs to properly identify the candidates of future paradigms. Table 2 shows Top 10 PP in each period, and the two previous paradigmatic patents whose GP = 1.0 were successfully identified and almost 70% of other identified patents are also ranked in every period. This can support that PP needs only next few years for identification. We qualitatively analyzed Top 10 PP patents and found some patents which are not only relatively new but also technologically promising.

**Table 2:** Top 10 PP patents.

Next 5 years		Next 10 years		Next 15 years	
Patent #	PP	Patent #	PP	Patent #	PP
US 5925525	2.07	US 4683195*	3.1	US 4683195*	3.61
US 6190857	1.87	US 5925525	2.4	US 5925525	2.27
US 6054270	1.11	US 6190857	2.09	US 5202231	2.18
US 4683195*	1.01	US 6054270	1.88	US 6054270	2.16
US 4358535*	1.0	US 5202231	1.66	US 6190857	1.97
US 5866336	0.99	US 5866336	1.31	US 4683202	1.87
US 5801154	0.79	US 4683202	1.13	US 4395486*	1.68
US 6251639	0.74	US 4358535*	1.12	US 4683194	1.52
US 5270163	0.64	US 5744305	1.06	US 5866336	1.52
US 5744305	0.55	US 4965188	1.0	US 4358535*	1.51

*: previous paradigmatic patents

US 5925525 (Top 1 by PP) provides compounds of a physical substrate called DNA microarray and reagents for hybridization. Such DNA chips are suitable for high-throughput sequencing and enable to automatically attach reagents to target nucleotides by attaching plural reagents which cover all possible length of nucleotides over 5 to a substrate. This invention is one of basic technology for very large-scale immobilized polymer synthesis system and so it has high possibility to become the future paradigm.

US 6251639 (Top 8 by PP) provides a new isothermal, linear amplification method. This patent amplifies both target polynucleotide sequence and polynucleotide sequence complementary to a target. Through the two amplification, the result RNA does not contain 3’ end sequence and it protects from contamination of RNA generated by PCR. In particular, this invention is distinguishable from other amplifications because of the non-contamination feature. Since the invention proposes a different approach to solve the contamination problem, it has high potential to be the breakthrough knowledge in the TD.

US 6054270 (Top 3 by PP) invented a method for chemically synthesizing a high-density array of oligonucleotides of chosen monomeric unit length within discrete cells or regions of a support material. This invention proposed a new concept of large-scale deposition of oligonucleotide sequences on a physical substrate and, in particular, it covers attaching synthesized oligonucleotides to a solid support, such as a glass plat or film, containing an array of oligonucleotides to identify DNA sequences, under hybridization conditions [40]. Therefore, it has been recognized as one of the fundamental inventions regarding the manufacture and use of DNA microarrays that enable high throughput sequencings with effective way [41].

**Fig 8:**
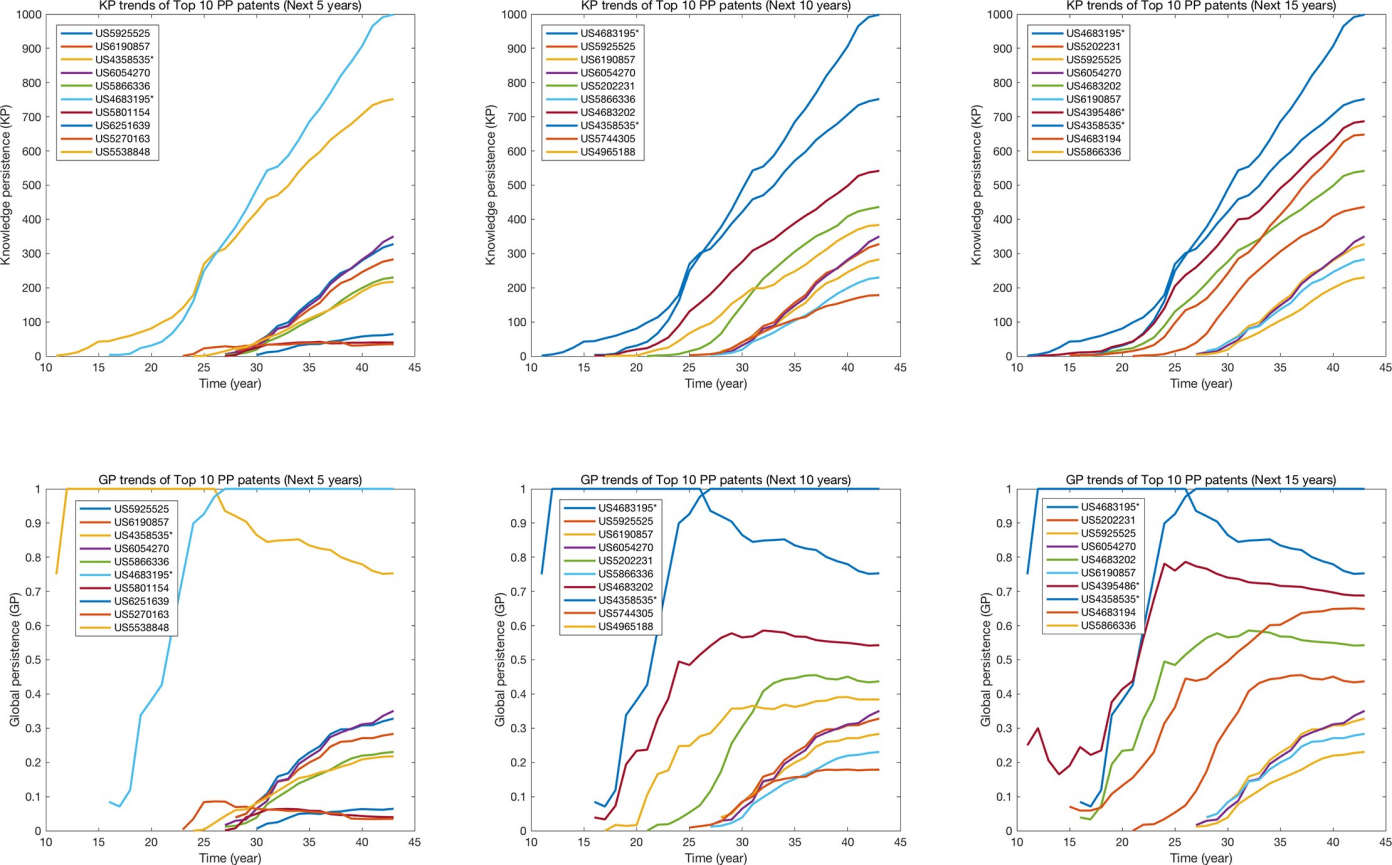
Top 10 PP patents in the next 5, 10 and 15 year period. *Note*: ‘*’ denotes previous paradigm patents.

Even though our qualitative analysis shows that PP well identifies the candidates of future paradigms, Fig 8 and Table 2 show that some late-blooming inventions, such as US 4395486, one of previous paradigms, require a longer time to be identified by PP, because their gradient in knowledge persistence in the early years might not be higher than others. Therefore, qualitative analysis for the PP results should be supplemented not only to avoid missing important inventions, but also to increase the reliability of the results.

## Conclusion

This paper proposes a method to quantitatively identify dynamic changes of technological paradigms. Specifically, the method identifies previous paradigms and candidates of future paradigms in a specific TD. To identify paradigmatic inventions which dominantly influence on the recent technological developments, we adopted a knowledge persistence measurement [18] that quantifies the degree of technological of a patent on the recent inventions by measuring the amount of remained knowledge of the patent in the most recent patents. The knowledge persistence of a patent changes over time; knowledge persistence of a patent is dramatically increasing when the patent dominantly impacts on later inventions, but knowledge persistence is decreasing (or maintained) if the patent is no longer important in a TD. This changing trends shows knowledge persistence can represent relative importance of patents in different timeframe. Therefore, the top knowledge persistence patents in specific periods can be represented as technological paradigms and the changes of them over time show the trends in paradigm shifts. We developed a new metric (PP) to identify the candidates for future paradigms which are basically difficult to be identified by using citation-based indicators. The main concept of the PP is based on the increasing trends of the previous paradigms’ knowledge persistence over time and the PP can identify the recent patents having low knowledge persistence but high possibility to become future paradigms in a TD. We conducted an empirical analysis using patents on Genome sequencing technology. The all identified previous paradigms and candidates for future paradigms are qualitatively analyzed. In particular, the results that all previous paradigms were also identified as the top ranking by PP can support the reliability of the future paradigms by PP.

However, some issues should be supplemented or improved in the further work. First, use of both patent applications and granted patents might provide better results for future prediction. This research only used the granted patents as an input data, and so the number of forward citations of late patents in our patent set is not good enough. Therefore, use of patent applications as a supplement data can provide better results in PP analysis. Second, as mentioned in Section 4.3, the PP might need long period to identify the late-blooming inventions. Considering that knowledge persistence of most paradigmatic inventions increases dramatically fast from their emergence, this limitation might not be a big problem. But there still has possibility, like US 4395486 case, that some paradigmatic inventions have the characteristic of late-blooming invention. Therefore, further research will improve predictability of PP by considering other important indicators, such as knowledge diffusion trend or topological structure of forward citations in a patent citation network, and extraction of novel technological knowledge by text mining technique. Third, although this paper identifies potential candidates of future paradigms, other predictions on future paradigms, such as the estimated knowledge persistence of a patent in a specific future time, or the timing of paradigm shifts in the future, can increase richness of the method. The recent deep neural network technique with a large number of learning samples of HPPs from all US patents seems to be a doable approach for the purpose. Lastly, since the proposed method needs only patent (backward) citation data in a TD, it is applicable to any different TDs without additional revisions. However, there is possibility that the method might provide different results, if knowledge diffusion (i.e. citations) trend in a TD is very different from most of TDs. Therefore, our further works will include applications of the method to many different TDs.
